# Na/K-ATPase Signaling and Salt Sensitivity: The Role of Oxidative Stress

**DOI:** 10.3390/antiox6010018

**Published:** 2017-03-02

**Authors:** Jiang Liu, Yanling Yan, Ying Nie, Joseph I. Shapiro

**Affiliations:** 1 Department of Biomedical Sciences, Joan C. Edwards School of Medicine, Marshall University, Huntington, WV 25755, USA; liuj@marshall.edu (J.L.); Yan@marshall.edu (Y.Y.); Niey@marshall.edu (Y.N.); 2 Department of Medicine, Joan C. Edwards School of Medicine, Marshall University, Huntington, WV 25701, USA

**Keywords:** Na/K-ATPase, signaling, cardiotonic steroids, ROS, renal sodium handling, salt sensitivity

## Abstract

Other than genetic regulation of salt sensitivity of blood pressure, many factors have been shown to regulate renal sodium handling which contributes to long-term blood pressure regulation and have been extensively reviewed. Here we present our progress on the Na/K-ATPase signaling mediated sodium reabsorption in renal proximal tubules, from cardiotonic steroids-mediated to reactive oxygen species (ROS)-mediated Na/K-ATPase signaling that contributes to experimental salt sensitivity.

## 1. Introduction

According to the 2016 American Heart Association (AHA) Statistical Update [1], 32.6% (about 80 million) of people in the United States aged 20 and older have hypertension, and about 54.1% of those cases have their hypertension well-controlled. Hypertension is a major risk factor for cardiovascular events and mortalities. The development and maintenance of hypertension is complicated. The causes are still not well defined in most hypertension cases (about 90%–95%). In the 2008 AHA Scientific Statement [2], excessive dietary salt ingestion contributes to the development and maintenance of hypertension and tends to be more pronounced in older salt-sensitive people, African Americans, and those with chronic kidney diseases [3,4,5]. In the 2016 AHA Scientific Statement [6], salt sensitivity was defined as a physiological trait by which blood pressure (BP) changes are parallel to changes in salt intake. In human beings, salt sensitivity is “a continuous, normally distributed trait”. United States Department of Agriculture (USDA) Guidelines and AHA Scientific Statements all suggest that a modest dietary salt reduction along with a balanced diet would be beneficial in BP control.

Collectively, there are considerable amount of factors that regulate both BP acutely and chronically. One major regulating factor is the kidney, which regulates systemic body fluid composition and volume. Early studies with kidney cross-transplantation showed that “hypertension goes with kidney” [7,8,9,10,11]. It is well accepted that renal sodium handling is a key determinant of long-term BP regulation [2,12,13,14]. Along the nephron, renal proximal tubules (RPTs) claim over 60% reabsorption of filtered sodium, which plays a critical role in the pathogenesis of salt-sensitive hypertension in humans [15,16,17,18,19,20,21] and in animal models, including spontaneously hypertensive rats (SHR) [22,23,24], Dahl salt-sensitive rats [25,26] and Milan hypertensive rats [27].

## 2. The Concept of “Natriuretic Hormone” and Renal Sodium Handling

The concept of “natriuretic hormone” or “third factor” eliminating excessive sodium by direct inhibition of Na/K-ATPase was proposed decades ago [28,29,30,31]. The concept of the “natriuretic hormone” proposed that excessive dietary salt intake increases circulating endogenous digitalis-like substances that are specific inhibitors and ligands of Na/K-ATPase. These substances can directly inhibit renal tubular Na/K-ATPase, leading to inhibition of renal sodium reabsorption and an increase in natriuresis to correct volume expansion and related BP increase. Accumulating evidence supports this concept under pathophysiological conditions such as high salt intake, chronic renal failure and chronic heart failure in various animal models and human beings [30,32,33,34,35,36,37,38,39,40,41,42,43,44,45,46,47,48]. The endogenous digitalis-like substances, also known as cardiotonic steroids (CTS), were recently identified as a new class of endogenous steroid hormones whose production and secretion are regulated by many factors including angiotensin II, adrenocorticotropic hormone, high salt intake, volume expansion, chronic renal failure and congestive heart failure [48,49,50,51,52,53]. Since the rodent Na/K-ATPase α1 subunit is much more ouabain-resistant (insensitive to ouabain treatment) compared to other ouabain-sensitive species such as dog, sheep and pig, the ouabain-induced natriuretic effect is more profound in ouabain-sensitive species. Gene replacement studies (in which the ouabain-resistant mouse Na/K-ATPase α1 subunit was replaced with ouabain-sensitive humanized α1 subunit) have unequivocally demonstrated an important role of endogenous CTS in the regulation of renal sodium excretion and BP in rodents [54,55]. CTS also cause significant oxidative stress and cardiovascular remodeling independent of their effect on BP [56,57,58,59].

Renal sodium reabsorption is controlled by many hormones and neurotransmitters, including parathyroid hormone (PTH), dopamine, epinephrine and norepinephrine, angiotensin II, insulin and glucocorticoids (reviewed in [60]); Na/K-ATPase is the driving force by coupling with the rate-limiting sodium-hydrogen exchanger (Na^+^/H^+^ exchanger isoform 3, NHE3). Accumulating evidence indicates that the ion transport capacities (activities) of the Na/K-ATPase and NHE3 are mainly regulated through protein trafficking under different conditions [61,62,63,64,65].

## 3. The Role of CTS in Na/K-ATPase Signaling Mediated RPT Sodium Handling

Ouabain (CAS# 630-60-4, C_29_H_44_O_12_) is a plant-derived CTS that can be found in *Acokanthera schimperi* and *Strophanthus gratus* plants. Ouabain was also isolated and identified in human plasma as an “endogenous hormone” [49,66]. With the discovery that the signaling function of Na/K-ATPase is stimulated by low doses of ouabain, we investigate the Na/K-ATPase signaling-mediated renal sodium handling and its role in salt-sensitive hypertension. Na/K-ATPase signaling has been widely studied over almost two decades and has been discovered and verified in different cells, tissues, organs and whole bodies. The topic has also been extensively reviewed. In essence, CTS bind to the Na/K-ATPase α1 subunit (reviewed in [67]) and activate the α1 subunit-associated c-Src (which interacts with the Na/K-ATPase α1 subunit to form the receptor Na/K-ATPase/c-Src complex), resulting in the stimulation of multiple protein-protein interactions and multiple protein kinase cascades, including, but not limited to, c-Src kinase, extracellular signal-regulated kinases 1 and 2 (ERK1/2), phosphoinositide-3 kinase (PI3K) and protein kinase C (PKC), as well as increased reactive oxygen species (ROS) production in both in vitro and in vivo models (reviewed in [32,35,36,48,52,68,69,70,71,72,73,74,75,76,77]). Even though the mechanism and the specific species of ouabain-stimulated ROS still remain unclear, our observations suggest that ouabain stimulates Ras-dependent, and at least in part, mitochondria-originated superoxide anion/hydrogen peroxide production [78,79,80]. In this review, we use the general term “ROS” to express ouabain-stimulated ROS increase. Increases in superoxide anion/hydrogen peroxide and reactive nitrogen species nitric oxide, which are mainly produced by mitochondria, NADPH oxidase, xanthine oxidase and uncoupled NO synthesis, can modify the Na/K-ATPase α and β subunit to inhibit the Na/K-ATPase enzymatic (ion exchange) activity [81,82]. However, while ouabain has been shown to induce mitochondria-related ROS generation [78,83], the role of NADPH oxidase, xanthine oxidase and uncoupled NO synthesis in the regulation of Na/K-ATPase signaling function is not clear. We will discuss the effect of H_2_O_2_ on Na/K-ATPase signaling and carbonylation modification in detail later. At the low doses without significant acute inhibition of the Na/K-ATPase ion-transport activity, ouabain-stimulated Na/K-ATPase signaling is largely independent of the changes in intracellular Na^+^ and K^+^ concentration activity demonstrated in rat neonatal myocytes and pig renal proximal tubule cell lines [78,84,85].

Using pig RPT cell line LLC-PK1 cells as a model, we found that a low concentration of ouabain induces significant depletion of the basolateral membrane-bound Na/K-ATPase and decreases in transcellular ^22^Na^+^ transport without intracellular Na^+^ changing [78,86,87]. Specifically, ouabain stimulates Na/K-ATPase/c-Src signaling, leading to caveolin-1-dependent and clathrin-dependent endocytosis of the Na/K-ATPase α1 subunit, a process mediated by activation of c-Src and PI3K (phosphatidylinositol 3-kinase) [87,88]. Moreover, in male Sprague Dawley rats, a high-salt diet (4% NaCl) increased urinary sodium and marinobufagenin (MBG, another cardiotonic steroid) excretion, reduced proximal tubular Na/K-ATPase enzymatic activity and induced endocytosis of the proximal tubular Na/K-ATPase α1 subunit. These effects were significantly attenuated by administration of an anti-MBG antibody [89], suggesting that redistribution of Na/K-ATPase in RPT epithelium in response to endogenous CTS might play an important role in renal adaptation to salt loading.

With normal renal function, renal adaptations to volume expansion and dietary sodium intake involve a marked downregulation of RPT sodium reabsorption. The Na/K-ATPase endocytosis and reduction of transcellular Na^+^ transport without changes in intracellular Na^+^ concentration suggest that ouabain might cause a physiological reduction of transcellular Na^+^ reabsorption. For this mechanism to function in the physiological setting, however, the apical component of proximal tubule Na^+^ reabsorption would also have to be negatively affected by ouabain. In polarized RPTs, apical Na^+^ entry is mainly represented by NHE3, an apical membrane-bound sodium-hydrogen exchanger [90]. NHE3 provides a critical mechanism for Na^+^, HCO_3_^−^ and fluid reabsorption in the RPTs [91,92]. NHE3 knockout mice showed reduced RPT Na^+^ and HCO_3_^−^ reabsorption with urinary Na^+^ and HCO_3_^−^ wasting [91]. While increases in BP redistributed RPT NHE3 [93,94], chronic salt loading downregulated RPT NHE3 [95]. Trafficking of NHE3 is regulated by changes in endocytosis and/or exocytosis [96,97,98,99]. In epithelial cells, lipid rafts are involved in c-Src-dependent regulation of NHE3, both in signaling and trafficking [100]. Depressing apical Na^+^ entry without hypertension is not sufficient to decrease Na/K-ATPase activity, and depressing Na/K-ATPase activity alone is not sufficient to decrease RPT Na^+^ reabsorption; thus, it was proposed that coordinated decreases in both NHE3 surface distribution and Na/K-ATPase activity may be important for the response to hypertension [65,101,102,103].

We have demonstrated that ligand-mediated RPT Na/K-ATPase/c-Src signaling regulates RPT sodium reabsorption, and impairment of Na/K-ATPase/c-Src signaling contributes to experimental salt-sensitive hypertension [26,85,86,88,89]. In LLC-PK1 cells, ouabain activates the Na/K-ATPase/c-Src signaling pathway and coordinately stimulates the redistribution of basolateral Na/K-ATPase and apical NHE3, which is dependent on c-Src, PI3K and caveolin-1. This regulation, without influencing intracellular sodium concentration, leads to reduced transcellular ^22^Na^+^ transport in RPTs. When this regulatory mechanism was tested in vivo in Dahl salt-sensitive rats and salt-resistant rats fed a high-salt diet, the salt-resistant rats showed reduced sodium reabsorption and increased total urinary and RPT-mediated fractional sodium excretion with the activation of Na/K-ATPase signaling (interaction between the Na/K-ATPase α1 subunit with c-Src, activation of c-Src and ERK1/2) and redistribution of the Na/K-ATPase and NHE3 in isolated RPTs. However, the aforementioned observations in the salt-resistant rats were not seen or were significantly less in the salt-sensitive rats with raised BP. Moreover, this mechanism was also shown in Sprague Dawley rats [26,89]. This indicates that, in response to salt loading, activation of Na/K-ATPase signaling is beneficial in the regulation of RPT sodium handling, leading to salt resistance, and impairment of the signaling contributes to salt-sensitive hypertension.

## 4. The Role of ROS in Na/K-ATPase Signaling Mediated RPT Sodium Handling

The different response of the Na/K-ATPase signaling to salt loading between the salt-resistant and salt-sensitive rats could not be simply explained, since both strains have the same sequence of the Na/K-ATPase α1 subunit (the predominant α isoform in RPTs) and the same content of salt loading. This raised the question that other factor(s), except CTS, might activate or influence the Na/K-ATPase signaling. We have observed that ouabain-stimulated Na/K-ATPase signaling increases ROS generation, and pretreatment with antioxidants blocks the effects of ouabain [84]. ROS stress is both a cause and a consequence of hypertension [104,105,106,107,108]. In kidneys, physiological ROS functions as an important second messenger; but increased ROS influences RPT sodium handling [109,110,111,112], and pathological ROS stress causes renal dysfunction [113,114]. In different animal models, administration of antioxidants exhibits a beneficial effect on BP control (reviewed in [106]). In RPTs, increases in ROS stress activate Na/K-ATPase signaling, leading to the redistribution of Na/K-ATPase and NHE3 from membrane to cytosolic endosomes, which in turn reduces the capacity of sodium transport across the RPTs by the reduction of sodium entry through apical NHE3 and extrusion through basolateral Na/K-ATPase. This process reduces the sodium reabsorption and contributes to the development of salt-sensitive and volume expansion-mediated hypertension [109,110,112,115,116,117,118,119,120,121].

Interestingly, both Na/K-ATPase and Src protein tyrosine kinases are redox-sensitive [122,123,124,125,126,127]. We have demonstrated that a sustained non-toxic level of H_2_O_2_ promotes ouabain-induced cellular changes and endocytosis of Na/K-ATPase [128]. On the other hand, ROS stimulated by ouabain functions as a downstream effector of Na/K-ATPase signaling [78,79,84,128]. We hypothesized that Na/K-ATPase signaling is redox-sensitive, whereby ROS production induced either by CTS through the Na/K-ATPase α1 subunit or by other stimuli is necessary for its signaling and the subsequent inhibition of RPT sodium reabsorption (reviewed in [129]). However, chronic ROS overstimulation may overstimulate oxidative modification of the Na/K-ATPase α1 subunit, rendering desensitization of Na/K-ATPase signaling and, thus, dysregulation of RPT sodium reabsorption and BP.

In LLC-PK1 cells, ROS is clearly involved in the regulation of Na/K-ATPase signaling and RPT sodium handling. An increase in ROS by glucose oxidase activates Na/K-ATPase/c-Src signaling and redistributes Na/K-ATPase and NHE3, leading to inhibition of active transepithelial ^22^Na^+^ transport from the apical to basolateral aspect. Ouabain-induced activation of Na/K-ATPase signaling requires a basal physiological range of ROS since blockage of ROS prevents the initiation of ouabain-Na/K-ATPase signaling function. These observations suggest that ouabain-induced ROS generation further stimulates Na/K-ATPase signaling through a positive feedback mechanism, as well as that ROS is essential in initiating Na/K-ATPase signaling, and sufficient antioxidant capacity can prevent Na/K-ATPase signaling [80]. In LLC-PK1 cells, both ouabain and glucose oxidase stimulate direct protein carbonylation of the Pro222 and Thr224 amino acid residues of the Na/K-ATPase α1 subunit [80,130]. It has been demonstrated that protein carbonylation can function as a means of signal transduction, showing that ligand-receptor-mediated signaling promotes ROS-dependent protein carbonylation and proteasome-dependent degradation of carbonylated proteins [131,132]. A single mutation of Pro222 to Ala (Pro224Ala in rat α1) does not affect ouabain-induced inhibition of Na/K-ATPase enzymatic activity and ion-exchange activity, but abolishes the effects of ouabain on Na/K-ATPase/c-Src signaling, carbonylation modification, Na/K-ATPase endocytosis and active transepithelial ^22^Na^+^ transport. The data indicate that direct carbonylation modification of rat α1 Pro224 determines ouabain-mediated Na/K-ATPase signal transduction and subsequent regulation of RPT sodium transport.

In rodents, infusion of CTS and renal 5/6th partial nephrectomy cause ROS stress and protein oxidation in experimental animals [58,133]. Neutralization of CTS by administration of antibody against marinobufagenin (MBG) has been shown to reduce natriuresis in response to a high salt diet [89]. Renal 5/6th partial nephrectomy and a high fat diet are known to induce oxidative stress in rodents, leading to the development of experimental uremic cardiomyopathy and adipogenesis, respectively. Recently, we have demonstrated that both manoeuvers activate Na/K-ATPase/c-Src signaling and cause carbonylation modification of proteins. Administration of pNaKtide (a peptide originated from a segment of the Na/K-ATPase α1 subunit, functioning as an antagonist of Na/K-ATPase/c-Src signaling), in both cases, blocks the signaling and carbonylation modification, as well as significantly attenuates the phenotype of uremic cardiomyopathy by partial nephrectomy and adipogenesis by a high fat diet [134,135].

## 5. The ROS Paradox

In Dahl salt-sensitive rats, salt induces increases in aortic superoxide (O_2_^•−^) mainly through an angiotensin-dependent NADPH oxidase pathway; however, blockage of O_2_^•−^ does not lower the salt-induced increase in systolic BP [136,137].

In human beings, elevated endogenous CTS has been implied in aging-related salt sensitivity [138] and about 50% of untreated essential hypertensive patients [139]. Human studies seem to support a role of oxidative stress in the development of hypertension [104,106]. Hypertensive patients have increased levels of oxidative stress byproducts together with lowered activity of endogenous antioxidant enzymes in blood and mononuclear cells [140]. In comparison to control normotensive patients, hypertensive patients have been shown to have a reduction in superoxide dismutase and glutathione peroxidase activity, higher superoxide and hydrogen peroxide, which returned to control levels after BP reduction, and higher lipid hydroperoxide production [141,142,143,144]. The Dietary Approaches to Stop Hypertension (DASH) study and subsequent studies demonstrated that dietary pattern and salt intake interventions lower BP, which is related to reducing oxidative stress and reducing dietary salt intake [145,146,147,148].

## 6. Conclusions and Perspective

**Figure 1:**
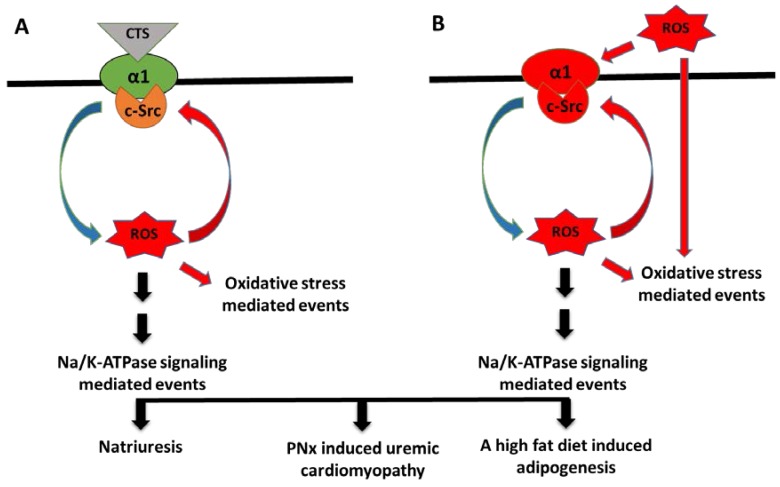
Illustration of the Na/K-ATPase signaling-mediated oxidant amplification loop, showing increases in cardiotonic steroids (CTS) (**A**) or increases in ROS within physiological range (**B**); stimulated oxidant amplification loop. Increases in CTS alone or increases in ROS alone or a combination of increases in CTS and ROS (not shown) stimulate Na/K-ATPase/c-Src signaling to induce Na/K-ATPase signaling-mediated events, such as renal sodium handling in proximal tubules (natriuresis), PNx-induced uremic cardiomyopathy and a high fat diet-induced adipogenesis.

Based on the observations of the interplay amongst CTS, ROS and Na/K-ATPase signaling, we have proposed that Na/K-ATPase, along with its ion-pumping and signaling function, also functions as an amplifier/receptor for oxidant signals, by coupling with c-Src (the Na/K-ATPase/c-Src signaling complex) (Figure 1). While Na/K-ATPase is the specific target of CTS, oxidative stress and CTS-induced increases in ROS can also target systems other than the Na/K-ATPase/c-Src complex. Activation of this oxidant amplification loop would generate more and more ROS, raising ROS from the physiological to the pathophysiological level, which will lead to over-stimulation of Na/K-ATPase/c-Src signaling, which in turn, renders the signaling complex inactive to further stimulation. This might also be the case of chronic oxidative stress in which Na/K-ATPase signaling is insensitive to stimulation. By interrupting this amplification loop, pNaKtide antagonizes Na/K-ATPase signaling-mediated oxidant amplification and oxidative modification of Na/K-ATPase and other proteins, to relieve the overall oxidant stress to the physiological level that will restore the capability of CTS-mediated Na/K-ATPase signal transduction [134,135]. On the other hand, in clinic trials, antioxidant supplementation has been shown to be beneficial (such as combination antioxidant supplement containing zinc, ascorbic acid, α-tocopherol and β-carotene, as well as glutathione and vitamin C) [149,150,151], ineffective (such as a combination antioxidant supplement containing ascorbic acid, synthetic vitamin E and β-carotene) [152] or even dangerous [106,153,154]. Using general antioxidants might not be effective to bring oxidative stress to a physiological level (with lower doses of antioxidants) or might totally naturalize ROS and bring cells into a sub-physiological level or even a reducing state (with high overdoses of antioxidants) that will affect many normal physiological functions at the cell/tissue/organ levels. There is no doubt about the association of food and health. The U.S. Department of Agriculture/Department of Health and Human Services 2010 Dietary Guideline for Americans (available at https://health.gov/dietaryguidelines/) indicates that certain supplements may be beneficial for certain special populations with some chronic diseases, but excessive usage of certain supplements might be harmful. The impact of the usage of multiple vitamins and minerals in individuals needs to be evaluated based on the individual’s health condition, life style, genetics and other factors [155].

Our observations indicate that the redox-sensitive Na/K-ATPase signaling contributes to salt sensitivity by regulating sodium handling in renal proximal tubules. Targeting the Na/K-ATPase signaling-mediated oxidant amplification loop might have therapeutic implications. Nevertheless, the mechanism is still not totally clear and needs to be further investigated.
